# Feasibility of a physical exercise intervention for patients on a palliative care unit: a critical analysis

**DOI:** 10.1186/s12904-024-01388-5

**Published:** 2024-02-28

**Authors:** Inken Schwonke, Nils Freitag, Paula Aschendorf, Kerstin Wucharz, Johanna Thieme, Iris Appelmann, Moritz Schumann, Frank Elsner

**Affiliations:** 1 Department of Palliative Medicine, Medical Faculty, RWTH Aachen University, Aachen, Germany; 2 Olympic Training Centre Berlin, Berlin, Germany; 3 Department of Molecular and Cellular Sports Medicine, Institute of Cardiovascular Research and Sports Medicine, German Sport University Cologne, Cologne, Germany; 4 Department of Physiotherapy, Franziska-Schervier Educational Center, Bethlehem Hospital, Stolberg, Germany; 5 Pro Sanum Health and Therapy Center Eifel, Nettersheim, Germany; 6 Department of Physiotherapy, RWTH Aachen University Hospital, Aachen, Germany; 7 Department of Sports Medicine and Exercise Therapy, Chemnitz University of Technology, Chemnitz, Germany

**Keywords:** Palliative care, Exercise training, Sports, Physical function, Quality of life

## Abstract

**Background:**

Recent exercise intervention studies have shown promising results in improving quality of life (QoL) and physical function (PF) in diverse chronic disease and advanced cancer patients. However, the effects of structured exercise in palliative care patients, having different therapeutic needs, lower life expectancies and PFs remain unknown. This study primarily aimed to assess the feasibility of an exercise intervention with follow-up by analysing recruitment numbers, screening procedures, acceptability, preferences, and safety of the exercise intervention as well as retention in follow-up. Our secondary aims related to changes in QoL and PF.

**Methods:**

This study comprised of a one-arm design without a control group. Over 6 months, every in-hospital palliative care unit (PCU) patient was screened for eligibility. Eligible patients were asked to participate in a 2-week exercise intervention consisting of resistance training and/or endurance training with moderate or high intensity based on personal preferences and a 4-week follow-up. Before and after the exercise intervention, QoL and PF were assessed and a qualitative interview after the intervention addressed expectations and experiences of the exercise intervention. For follow-up, patients were provided with information on independent training and after 1 and 4 weeks a QoL assessment and qualitative interview were conducted.

**Results:**

Of 124 patients screened, 10 completed the intervention with an adherence rate of (80 ± 25%), of which 6 patients completed follow-up. Endurance training was the most performed training type and only a few minor adverse events occurred in certain or likely connection to the exercise intervention. While physical QoL and PF measured by arm curl strength and time up and go performance improved, mental QoL and the other PF tests remained unchanged.

**Conclusion:**

Despite the challenges that were faced in our screening and testing process, that are specific to the palliative patient population with their unique therapeutic requirements and varying mental-/ physical capabilities, we discovered the 2-week exercise intervention to be feasible, safe, and well tolerated by palliative care patients. Moreover, it seems that short-term improvements in QoL and PF are possible. Further full scale studies are required to confirm our findings.

**Trial registration:**

The study was retrospectively registered on 25.01.2022 in the German Clinical Trials Register (DRKS00027861)

**Supplementary Information:**

The online version contains supplementary material available at 10.1186/s12904-024-01388-5.

## Background

Worldwide 56.8 million patients per year suffering from life-threatening diseases such as cardiovascular diseases or cancer require palliative care (PC) [1]. On a palliative care unit (PCU), treatment aims to stabilise patients with a limited life expectancy, focusing on symptom-control [2]. Current treatment recommendations already include supportive therapies (i.e. physiotherapy, psychotherapy etc.) on PCU´s to control individual symptoms, such as pain, fatigue and dyspnoea [2, 3]. The symptoms patients are facing affect the health-related quality of life (QoL), which can be self-assessed in terms of physical, psychological, and social aspects [3, 4]. However, at the end of life the assessment of QoL is complex due to expected physical and functional decline, and different targets of care [5]. Furthermore, PC patients often have a reduced mobility, leading to limited capacities to perform activities of daily living [6]. For example, on German PCU’s, over 78% of the patients have an Easter Cooperative Oncology Group (ECOG) performance status of 3 or higher [7], indicating a highly compromised physical function (PF; i.e. the capability to accomplish important activities for a self-determined life [8]). Previous work has shown that in advance cancer patients QoL correlates with PF [9] and that both can be improved by structured physical exercise [10], leading to overall improvements in activities of daily living.

Physical exercise has been shown to have beneficial effects in a variety of chronic diseases [11] and is safe even in advanced chronic conditions, such as cardiovascular disease [12], lung disease [13], multiple sclerosis [14], and chronic kidney disease [15]. For cancer patients, the guidelines of the American College of Sports Medicine recommend aerobic exercise of moderate intensity to be performed for a minimum of 150 minutes and strength training performed twice weekly [16]. Even though cancer morbidity and mortality rates depend on different cancer types and stages [17], physical exercise appears to improve the QoL, PF and social function also in advanced cancer patients [10, 18].

However, in contrast to PCU patients needing acute therapy for their underlying condition and symptom control, those patients included in previous studies generally had a good PF, a life expectancy of at least 6, but mainly 12 month or more, or were in a stable palliative care phase [3, 10, 19]. In turn, compared to most advanced cancer patients, PCU patients have different therapeutic needs and much lower life expectancies [5, 6, 7, 10, 19] but these patients appear to having been overlooked in the current exercise science literature. Interestingly, in a recent study 92% of patients with a life expectancy <12 months were interested in physical exercise [20] but with a lack of knowledge on the feasibility and preferences of such interventions it is difficult to cater to these needs. A few studies have assessed the feasibility of traditional physiotherapy on PCUs and hospices even in the last week of life [21–23]. However, in these studies the actual exercise interventions (if any) were not described in detail. Only 1 study in PCU patients showed promising results using gait training, stationary bike exercise and tilted table standing as well as bedside active and passive range movement [6]. Patients with good compliance of gym-based physiotherapy showed higher survival days [6]. However, an isolated feasibility and benefit analysis of a PCU exercise intervention study with defined training plans was not performed.

Considering the lack of knowledge concerning structured exercise interventions in PCU patients, the primary aim of this study was to assess the feasibility of a structured 2-week physical exercise program with 4-week follow-up for PCU patients by means of analysing recruitment and screening procedures, acceptability of the exercise programme and preferences of training types, retention of patients exercising during follow-up as well as safety [24]. As secondary outcomes, we were interested in the changes in QoL and PF during the in-hospital training program as well as changes in QoL during follow-up.

## Methods

### Study design

As this present study aimed at feasibility as a primary outcome, it included a single cohort without control group. Quantitative and qualitative methods were used to assess the primary and secondary outcomes and the consort statement information were used for reporting.

**Fig. 1:**
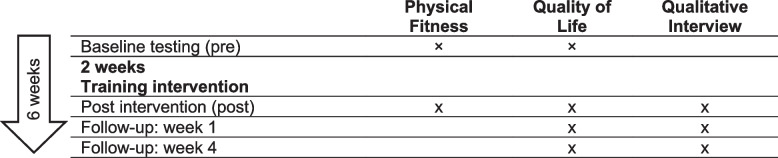
Study process for participating patients. The post intervention testing was scheduled 1 day before dismissal or after 2 weeks of the exercise intervention

The overall recruitment period was 6 months. Every patient admitted to the PCU was screened for eligibility. Eligible patients were asked to participate in an in-hospital exercise intervention for 2 weeks and encouraged to continue with self-directed exercise for a follow-up period of 4 weeks (Fig. 1). The intervention consisted of resistance (RT) or endurance training (ET) with moderate or high intensity. From Monday through Friday, patients were approached by physiotherapists and asked to select from a set of standardised exercise sessions. QoL and PF were assessed before (pre) and after (post) the 2-week in-hospital exercise intervention. In addition, a qualitative interview focusing on patients’ expectations and experiences of the exercise intervention was carried out after the intervention.

Following the intervention, patients were provided with information material on self-directed home-based exercise for the 2-week follow-up period (Additional files 1, 2). If the hospital stay exceeded the 2-week intervention time, PCU physiotherapist supported the self-directed training in the follow-up period. A follow-up QoL-assessment and qualitative interview were performed 1 and 4 weeks after the in-hospital intervention by phone or in person if dismissal was postponed.

### Participants

Patients admitted to the local PCU with an ECOG performance status of 1 to 3 [25] and an expected length of inpatient stay of approximately 2 weeks were eligible if no medical conditions prohibited study participation as assessed per treating physician. Exclusion criteria included uncontrolled high blood pressure and severe cardiac, orthopaedic, mental or sensory impairments. Patients with bone metastases were not generally excluded, but eligibility was discussed with physicians and physiotherapist on basis of existing exercise recommendations [26]. Written informed consent was provided before enrolment into the study.

### Exercise intervention

The exercise intervention was based on the individual ECOG performance status (Table 1, Additional file 3) and consisted of moderate or high intensity RT or ET as patients preferred. The intensity was defined by the rating of perceived exertion (RPE) on the modified BORG scale [27]. A RPE of 7-9 was required for high intensity and 4-6 for moderate intensity exercise, respectively [27]. ET was performed as hallway gait training, on a stationary cycling ergometer (ergoline; Bitz, Germany, ergoselect 1) or a hand crank ergometer (emotion fitness; Hochspeyer, Germany, Motion Body 600). RT consisted of different exercises for the upper and lower body, for which dumbbells (1-5 kg), loop bands and stepping boards were available (material list, Additional file 4). Each training session started and ended by a 3-minute low intensity exercise (RT: active and passive stretching, ET: training without load or joint mobilisation). Patients were approached and asked for the personal choice of exercise (ET or RT, moderate or high intensity). The corresponding type of training was then performed according to the individual ECOG performance status, state of disease and capabilities. In the presence of bone metastases, the exercise programme was further modified based on existing recommendations [26, 28]. Changes in the ECOG throughout the intervention were considered and documented.

**Table 1:** Training specifications based on the ECOG-status

**Endurance Training**												
	**ECOG 2**						**ECOG 3**					
**Intensity**	**Session number**	**Number of bouts**	**RPE**	**Exercise time [min]**	**Rest between bouts [min]**	**Total training time [min]**	**Session number**	**Number of bouts**	**RPE**	**Exercise time [min]**	**Rest between bouts [min]**	**Total training time [min]**
Moderate continuous	1-3	3	4-6	5	2	19	1-3	2	4-6	5	2	12
	4-6	4	4-6	5	2	26	4-6	3	4-6	5	2	19
	> 6	3	4-6	7	2	25	> 6	4	4-6	5	2	26
Intensive interval	1-3	6	7-9	0.5	1.5	12	1-3	4	7-9	0.5	1.5	8
	4-6	6	7-9	1	2	15	4-6	4	7-9	1	2	12
	> 6	6	7-9	1	1	12	> 6	4	7-9	1	1	8
**Strength Training**												
**Intensity**	**ECOG 2**						**ECOG 3**					
	**Session number**	**Number of exercises**	**RPE**	**Number of sets**	**Repetitions per set**	**Time between exercises/sets [min]**	**Session number**	**Number of exercises**	**RPE**	**Number of sets**	**Repetitions per set**	**Time between exercises/sets [min]**
Moderate	1-3	4	4-6	3	12-15	0.5	1-3	4	4-6	2	15-20	0.5
	4-6	6	4-6	3	12-15	0.5	4-6	6	4-6	2	15-20	0.5
	> 6	6	4-6	4	10-12	0.5	> 6	6	4-6	3	12-15	0.5
Intensive	1-3	4	7-9	3	8-10	0.5	1-3	4	7-9	2	8-10	0.5
	4-6	6	7-9	3	6-8	0.5	4-6	6	7-9	2	8-10	0.5
	> 6	6	7-9	4	3-5	0.5	> 6	6	7-9	4	6-8	0.5

In patients between 2 ECOG scores, the higher score was selected. The progression of the training load took place after every 3 sessions. It did not matter whether endurance or strength training took place

Time is presented in minutes [min]

*Abbreviations*: *ECOG* Eastern Oncology Group*, RPE* rating of perceived exertion

The number of training sessions offered and performed as well as reasons for not offering or performing sessions was recorded. Furthermore, type of exercise, equipment, duration, intensity (wattage or weight) and number of sets was recorded for ET and RT, respectively. To assess short-term changes in mood and constitution, patients were asked after each training session whether they felt better, same or worse compared to before the session.

## Outcomes

### Primary outcome: Feasibility

The feasibility analysis was based on Orsmond & Cohn [24] and consisted of the following aspects: recruitment and screening procedures, acceptability, retention, and safety.

For the evaluation of recruitment and screening procedures, we used data from the online University Hospital Aachen patient management system. Age, gender, date of admission and admission diagnosis were recorded. For eligible patients, we also collected dates of discharge from hospital, presence of bone metastases and days from admission to PCU until eligibility for participation. Acceptability and suitability of the intervention was evaluated by means of training protocols and interviews. Adherence rates were calculated by dividing the number of offered by the number of conducted training sessions. The different types of exercise (RT or ET, high and moderate intensity) were compared in terms of their frequency, completion rates, used equipment and individual mood states after each session. Retention of the intervention was assessed by recording reasons for drop-outs and number of training sessions performed during follow-up. Moreover, obstacles and personal wishes were included in the retention analysis. Safety was assessed by records of adverse (grade 1-2) or severe adverse events (grade 3-5) occurring during or in between training sessions, based on the Common Terminology Criteria for Adverse Events (CTCAE, Version 5 Published: November 27,2017) [29].

### Individual experiences, expectations, and barriers to physical exercise

Qualitative interviews were performed after the in-hospital intervention as well as during follow-up at week 1 and 4, using a semi-structured interview guide (full interview guides in Additional file 5). According to Dresing & Pehl [30], leading questions were formulated openly, text-generating, and simple. Rephrasing of questions or wording assistance in case of verbal difficulties or comprehension problems were possible. With patients’ verbal consent, interviews were recorded using a digital voice recorder. After the interviews, field notes were taken on the mood, interview situation, and patients’ condition (e.g. amnesic aphasia, concentration problems). The duration for each interview was 10 to 20 minutes. Questions were aimed at the role of sports and physical activities in patients’ lives, expectations and experiences with physical exercise, suggestions for improvement, and preferences for the type of exercise. Example questions included the following: What were your expectations of the exercise intervention? How did you like the exercise intervention? Have you felt any changes as a result of the exercise intervention?

Qualitative interviews were transcribed using MAXQDA (MAXQDA Plus 2022 Student, MQST22-EBBcmP-fPZ55A-14185h-E3YLLn) according to the simple transcription system of Dresing & Pehl [30]. Interviews were paraphrased and a category system was developed both deductively and inductively according to Kuckartz et al. [31]. For the post-intervention interview and for follow-up interviews a code tree was created respectively. A summary table, not including same content statements, was created.

### Quality of Life

QoL was assessed by the Short-Form-Health-Survey-12 (SF-12, 2^nd^ edition, time scale 1 week) through an interview. The questionnaire queries 8 dimensions of subjective health, to obtain a score for physical and mental QoL, respectively [32]. Scores for the physical summation scale and psychological scale were compared intra-individually.

### Physical fitness

Due to a lack of available fitness tests specifically designed for PCU patients, the German Olympic Sports Federation’s (DOSB) “Everyday Fitness Test” [33] was applied in a modified version. The test was originally designed for healthy elderly individuals and covers many important fitness components. The test equals the senior fitness test [34] and includes the following validated exercises: chair rise test, arm curl test, 2-minute leg raise test, timed up and go test, back stretch test and chair sit and reach test [33, 34, 35]. The order of testing was followed whenever possible. The following modifications were necessary: Weights were selected individually in 1kg steps, patients were allowed to use their usual assistive devices, exercises which were not possible due to the individual condition (e.g. bone metastases) were skipped [26]. Since we observed that the entire 6 tests were too strenuous for the patients in this study, the chair rise test was skipped when the time up and go test was possible. The weights used were kept similar in the pre and post-test. If post intervention testing was not possible due to adverse health conditions, this was recorded accordingly.

### Statistical analysis

Feasibility criteria were analysed by descriptive statistics. Changes in QoL and PF pre to post were assessed using Wilcoxon tests for non-parametric outcomes. The chair rise and chair sit and reach test were not evaluated due to insufficient data points. For patients completing the in-hospital intervention and follow-up, we evaluated changes in QoL using the Friedmann Test with Bonferroni correction. All tests were analysed with SPSS (IBM™, Version: 28.0.0.0). The level of statistical significance was set at *p* ≤ 0.05. Data are presented as mean ± standard deviation, unless indicated otherwise.

## Results

### Primary outcome

#### Recruitment characteristic and screening procedures

**Fig. 2:**
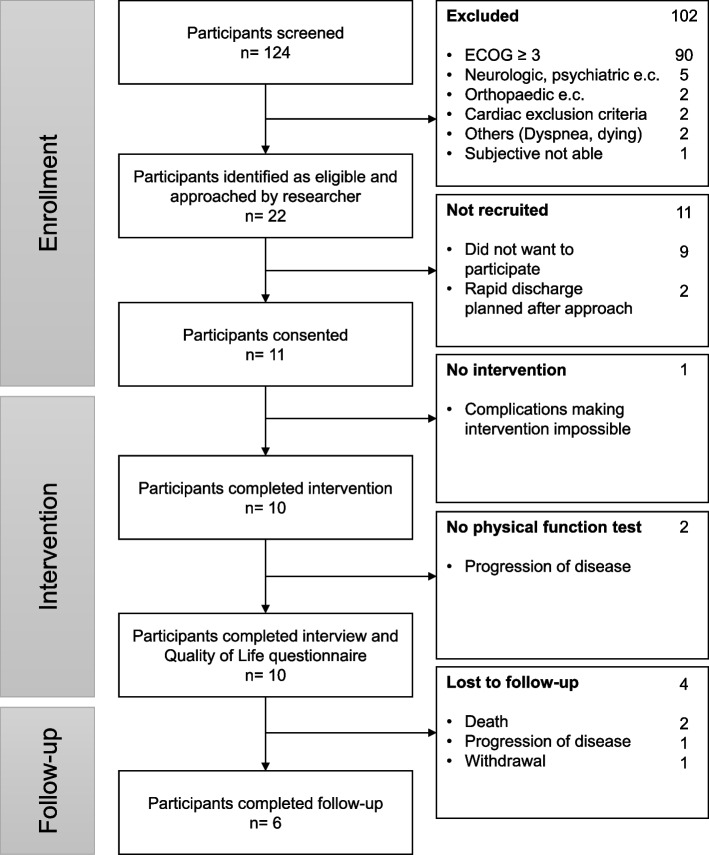
Patients-flow diagram shows the number of screened, recruited and participating patients throughout the 6-month period. Abbreviations e.c,: exclusion criteria Specifications of excluded patients Neurologic and psychiatric: dementia (3), psychooncological problems (1), hypokalemic paralysis (1) Orthopedic: instable bone metastases (2) Cardial: syncope (1), heart insufficiency (1)

After screening all patients in the time span of the 10/01/2022 until 20/07/2022 (Fig. 2), 22 patients were asked to participate in this study. Of those, 2 patients were rapidly (within 2-3 days) discharged and 9 patients did not want to participate. Reasons included lack of interest (*n* = 3), medical reasons (pain (*n* = 1), concerns about artificial joints (*n* = 1), a recent fall that had to be examined (*n* = 1)) and psychological or physical overload (*n* = 3).

After giving informed consent (Table 2), 11 patients participated in this study. The number of days from admission to enrolment differed from 1 to 35 days (9.5 ± 9.8). After enrolment, 1 patient could not train due to medical issues and did not complete the intervention. Similarly, 2 patients were not able to perform the PF-test due to disease deterioration.

**Table 2:** Patient characteristics

	**All patients**	**Eligible patients**	**Included patients**
n	124	22 (17.7%)	11 (8.8%)
Age (mean ± SD, range in yrs.)	69 ± 13 (33-91)	66 ± 11 (39-82)	64 ± 12.8 (39-77)
Women (n, %)	49 (40%)	10 (45.5%)	4 (36.5%)
Men (n, %)	75 (60%)	12 (55,5%)	7 (63,5%)
Cancer disease (n, %)	97 (78%)	21 (95.5%)	11 (100%)
Non-cancer diseases (n, %)	27 (22%)	1 (4.5%)	0
Bone metastases (n, %)		4 (18%)	3 (27%)
ECOG performance status			
1	0	0	0
2	4 (3%)	4 (18%)	2 (18%)
2,5/3	30 (25%)	18 (82%)	9 (82%)
Days as inpatient (mean ± SD, range in days)	11.8 ±12.9 (0-66)	25.1 ± 16.8 (7-66)	28.2 ± 9.9 (8-66)
Days admission to eligibility (mean ± SD, range in days)		8.3 ± 8.2 (1-35)	9.5 ± 9.8 (1-35)

The table shows the differences of all patients that were registered over the time in comparison to the eligible and included patients. Data are presented as number and percentage of the group or indicated otherwise

*Abbreviations*: *ECOG* Eastern Cooperative Oncology Group, *SD* standard deviation

### Acceptability and Suitability of Intervention and Study procedures

Of 67 training sessions offered across all patients, 70% were performed (Table 3). In 51% of the sessions and particularly frequent during gait training, training was not performed according to the plan. Either the number of sets or repetitions were not achieved or specified time intervals were not adhered to. Nevertheless, patients reported feeling subjectively better in 37%, 33%, and 25% of the moderate ET, intensive ET, and moderate RT sessions respectively. After 25% of the moderate RT sessions, however, patients felt worse. Overall, most patients felt the same after the sessions (62%), followed by better (36%) and worse (4%).

**Table 3:** Adherence to exercise intervention

Training monitoring parameters	Results
Offered sessions (n) Training monitoring parameters	61 Results
Conducted sessions (n)	47^a^
Adherence rate per person	80% ± 25% (25%-100%)^a^
Sessions per patient (mean ± SD, range)	4,7 ± 2,8 (1-8)
Days from inclusion to study until dismissal (mean ± SD, range)	18,7 ± 13,4 (2-50)
Type of exercise	
Moderate ET (n)	29
Intensive ET (n)	9
Moderate RT (n)	10
Intensive RT (n)	1
Session according to the plan (n, %)	23 (49%)
Session not according to the plan (n, %)	24 (51%)
ET (n, % like protocol)	38^a^ (39.5%)
Gait training (n, % like protocol)	28 (28.5%)
Hand crank ergometer (n, % like protocol)	10 (70%)
Bicycle ergometer (n)	0
RT (n, % like protocol)	11^a^ (73%)
Used weights	1kg-4kg
Used other material	Elastic band, easy loop band, stepper, body weight,
Not used	5-10kg weights, stronger loop bands, sling-trainer
Reasons for no training	
Weekend/ holiday (n)	32
Medical reasons (pain, dizziness, weakness, nausea) (n)	9
Not met by physiotherapist (n)	4
Physiotherapist in holiday or sick (n)	3
Need of rest (n)	5
Other (n)	5

^a^2 patients requested to perform both ET and RT in 1 training session and 1 patient trained once additionally voluntarily without a physiotherapist. For that reason, numbers differ between conducted sessions and the number of performed ETs and RTs

*Abbreviations*: *ET* Endurance Training, *RT* Resistance Training

In qualitative interviews, patients reported their expectations were fulfilled (Table 4) and they would recommend the exercise intervention to other patients. Though most experiences were positive, also negative aspects were mentioned (complete summary table in Additional file 6)

**Table 4:** Summary table of qualitative interviews post exercise intervention

**Subjects**	**Responses**
Expectations	Getting fitter More mobility Gaining strength
Valuation	8 patients valued therapy well to very well 1 patient did not like the exercise intervention 1 patient stated to benefit more from own movement
Positive experiences	Improvement of strength, mobility, stability Ability to walk or stand again Feeling physically and mentally better after the exercise intervention
Negative experiences	Muscle soreness Mental setbacks because something did not work out as expected Experiences of exceeding the physical limit
Changes through exercise	No changes in mood, fatigue, or pain as result of the intervention

### Safety

Overall, eight adverse events were reported having a likely connection, while 6 adverse events were unlikely connected to the exercise intervention (Table 5).

**Table 5:** Adverse Events

	**certain**	**likely**	**unlikely**
Grade 1	*n*=1 Fatigue	*n*=6 Fatigue, Dyspnoea, Flank pain, Myalgia Dizziness	*n*=2 Fall, Neck pain
Grade 2		*n*=2 Weakness upper limp, myalgia muscle	*n*=3 Constipation, stomach pain, Fatigue, Hyperglycemia
Grade 3			*n*=1 Pathologic humerus fracture

Data are presented as number and type of adverse events

Only one grade 1 adverse event had a certain origin in the intervention because fatigue occurred during the session and led to termination of this session. Additionally, fatigue and weakness during 2 moderate RT sessions led to a reduced training volume.

### Retention

**Fig. 3:**
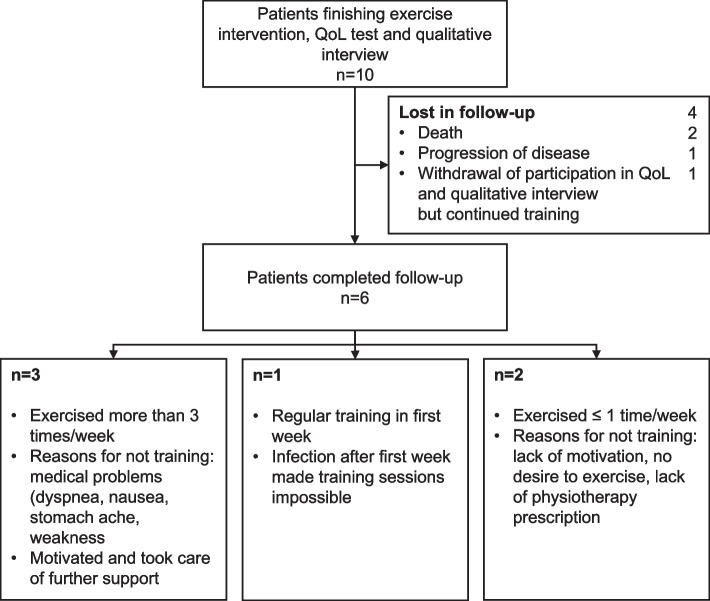
Follow-up patient flow with lost patients and self-reported numbers of training sessions as well as reasons for not training assessed through qualitative interviews. Abbreviations: QoL (Quality of Life)

Overall, 4 patients were lost during follow-up. The 6 patients completing the in-hospital intervention and follow-up period stated in the qualitative interviews that mainly medical problems prevented exercise training, while motivational aspects were also reported (Fig. 3, full follow-up summary in Additional file 7).

## Secondary Outcomes

### Quality of Life

**Fig. 4:**
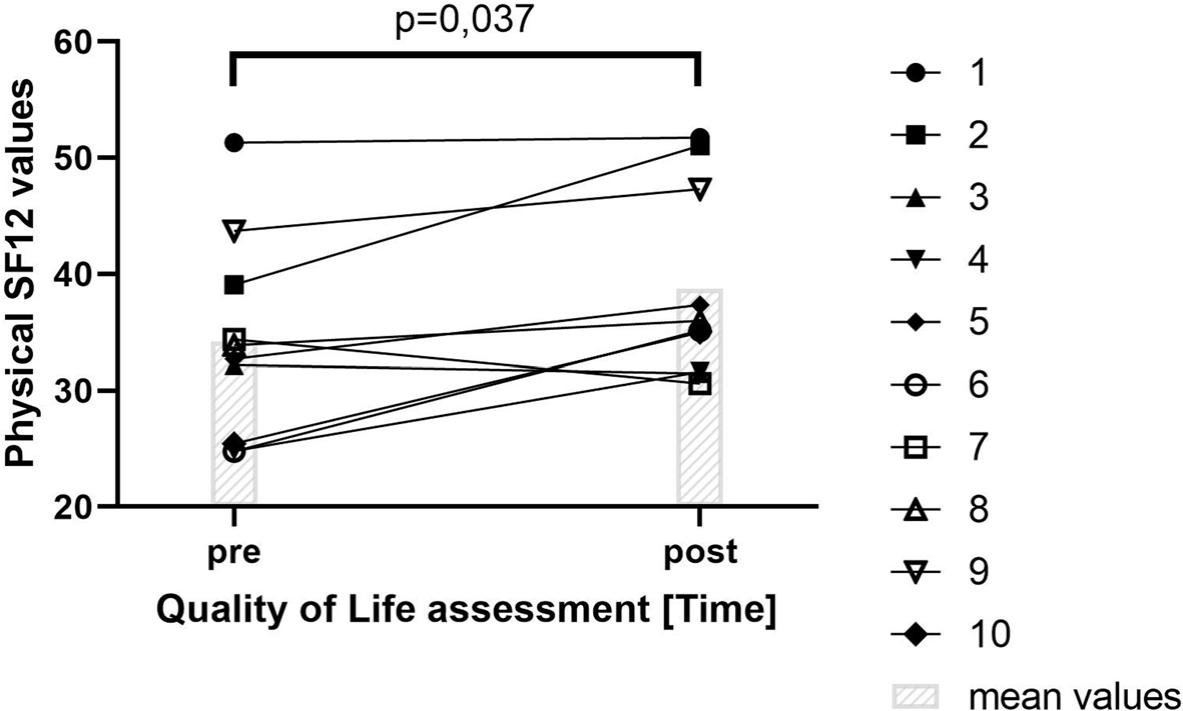
Changes in physical QoL assessed by the SF12 pre and post the 2-week exercise intervention.

The physical QoL statistically improved from 34.2 ± 8.6 to 38.7 ± 8.1 throughout the 2-week intervention (*p* = 0.037) (Fig. 4), while mental QoL remained statistically unchanged (55.6 ± 6.3 to 51.5 ± 13.2, *p* = 0.386).

Physical QoL of the 6 patients followed up for another 4 weeks showed a non-significant improvement pre to post in-hospital intervention but a statistically significant deterioration after the intervention to the 4-week follow-up (pre 34.5 ± 9.8, post 53.2 ± 6.2, 1 week 39.0 ± 8.9; 4 week 32.8 ± 11.5 *p*=0,044). Mental QoL remained statistically unaltered throughout the intervention and follow-up (before 41.0 ± 8.1, after 50.4 ± 13.7, 1 week 55.9 ± 5.3, 4 week 54.7 ± 10.4).

### Physical function

Arm curl strength (*n* = 8, 15.6 ± 4.2 repetitions to 19.0 ± 3.9 repetitions, *p* = 0.017) and time up and go performance (*n* = 6, 16.2 ± 13.8 sec to 12.2 ± 11.5 sec, *p* = 0.028) statistically improved throughout the 2-week intervention. Back stretch (*n* = 6, -31.9 ± 27.6 cm to -27.4 ± 25.2 cm *p* = 0.588) and 2-minute leg raise performance (*n* = 6, 39.0 ± 16.8 repetitions to 49.4 ± 24.3 repetitions *p* = 0.138) remained statistically unchanged.

## Discussion

The primary aim of this study was to assess the feasibility of structured physical exercise in PCU patients, while we were secondarily interested in the effects of exercise on QoL and PF. Even though we observed difficulties in recruiting patients, participating patients had a good adherence rate and preferred ET especially in moderate intensity during the in-hospital intervention. While only few minor adverse events occurred in relation to the in-hospital intervention, patients’ physical QoL statistically improved. The follow-up period identified medical problems and disease deterioration as the main barriers for continuing exercise training and may explain the physical QoL deterioration after 4 weeks follow-up.

### Recruitment capability and screening criteria

We screened a total of 124 patients in 6 months, equalling the average total number of patients in this period on the local 9-bed-PCU over the last 10 years (113.7 ± 11.8 patients). The screened patients stayed on average 11.8 ± 12.9 days (average last 10 years: 11.3 ± 1.3 days). While 22% were non-cancer patients, the majority of our enrolled patients were diagnosed with a cancer disease, which is common in German PCUs [7]. However, the functional status as assessed by ECOG was much lower than that observed in other PCUs [7]. While Brunner et al. [7] showed that 55% of PCU patients had an ECOG ≤ 3, in our study we observed that only in 28% of all patients and only in 19% of non-cancer patients. Especially non-cancer patients were often at the end of life or were not able to participate because of infections, fractures, or dementia. Nevertheless, approximately 10% of our PCU cancer patients were eligible and willing to participate. However, while it was previously reported that 92% of PC patients with a life expectancy between 3 to 12 months were interested and felt able to participate in physical exercise [20], we only found 50% (11 patients) of the eligible patients to be willing to participate in our study. Our screening criteria aimed to identify medical reasons that might compromise patient safety during training, while increasing the number of likely eligible patients. However, 6 of the 11 patients who declined participation stated that they did not want to participate for medical reasons (including psychological overload). Only 3 patients were simply not interested. This suggests our screening criteria were not sufficiently detailed.

The main exclusion criterion was an ECOG ≥ 4. The ECOG was used as screening criterion and outcome parameter in several exercise studies with advanced cancer and PC patients [36–40]. However, we observed difficulties with the ECOG as screening parameter. For example, patients’ ECOG changed over time but was not documented in the online system. Thus, some patients having an ECOG 4 on admission improved to ECOG 3 and were therefore eligible. Additionally, physicians had difficulties categorising patients in the ECOG system, resulting in 10 patients assessed with an ECOG between 3 and 4, for whom neither definite inclusion nor exclusion was possible and for non-cancer patients the ECOG is not applicable. There were also bedridden patients (ECOG 4) being able to train in bed but could not participate due to the high ECOG.

Furthermore, physicians had difficulties to ultimately decide whether diseases or mental impairment were too severe to participate. Especially the exclusion criterion “mental impairments” was difficult to assess. All 4 included brain tumour patients had some mental impairments but subjectively their PF was good. While patients with severe cognitive impairments were excluded, also minor cognitive impairment of included patients caused difficulties while testing. Additionally, opinions concerning eligibility differed between physicians and physiotherapists. Physicians were more critical about possibilities and benefits of exercise training than physiotherapists, highlighting the possible lack of awareness about benefits and risks associated with physical exercise.

### Acceptability of exercise intervention

The mean adherence rate to the intervention of 80% is within the range of 33-93% and higher than the median adherence rate of 69% stated in a previous systematic review on exercise interventions for advanced cancer patients [10, 19]. As expected for PCU patients, medical reasons were mainly responsible for patients’ refraining from participating in training sessions. Previous studies showed that rather classical physiotherapy (e.g. gait training, transfer training, not specified exercises, respiratory treatments) is feasible in the last week of life [22]. We clearly confirm these findings by also showing, that structured exercise training is feasible during the last weeks of life and improvements of QoL, and PF are possible.

In line with previous findings, it could be confirmed that gait training was the most preferred and performed type of ET [20]. For patients, gait training is directly correlated to their activities of daily living and easy to understand. Thus, patients see the need for gait training. Interestingly, during gait training we also found relatively the most deviations from the prescribed intervention, mainly due to exhaustion. Indeed, the planned training interval time of 5 minutes continuous gait training appeared to be too much for patients, as facilitation as with RT or with a hand-crank ergometer is not possible with a walker and the intensities can, thus, not be adjusted. Consequently, training with a hand-crank ergometer was well accepted by patients but caused logistic problems, as the device had to be transported by the physiotherapist to patients’ rooms. For patients this was often reason enough for choosing different training types as they thought it would be a burden for the physiotherapists. Nevertheless, in future studies we recommend using hand crank ergometer, recumbent ergometer and bed ergometer rather than seated ergometer and gait training in order to control and individualise the exercise intensities.

Concerning the training type, we saw a preference of ET in comparison to RT, supported by a higher rate of “feeling better” after ET sessions indicating a higher feasibility of ET. Nevertheless, RT was also possible and induced lower deviations from the training prescription. In terms of intensity, patients preferred moderate rather than high intensity. It should be noted, however, that the subjective control of intensity using the RPE scale presented difficulties. The RPE scale is a practical, affordable and valid option to monitor exercise intensity [41] and is commonly recommended for untrained individuals. As such it was used in a variety of athletic settings, elderly and cancer patients [41–43]. However, in our study the physiotherapists reported that patients experienced difficulties when reporting the perceived exertion by RPE. Possible reasons for that may be related to cognitive impairments due to drug side effects and a disturbed body perception caused by the rapid decline of PF due to hospitalisation and invasive medical treatments. These problems may also explain the preference to start with lower intensities as patients appeared to fear the experience of failure. In future studies, alternatives such as heart rate measurement or talk tests [44] may be considered to control intensity but these also require further assessment in terms of drug interactions and suitability in this particular population.

### Safety

No serious adverse events were observed in likely connection to the intervention. The most common adverse events having a likely or proven connection to the intervention were fatigue, weakness, and myalgia, however reversible by rest. Especially fatigue, though, is a common symptom in advanced cancer patients and often not completely reversible by rest [45]. Exercise has been found to be helpful for treatment [45] but in line with our findings and previous data, it may also acutely deteriorate fatigue [46]. In order to measure the effects of exercise in PCU patients on acute and chronic fatigue we recommend to asses fatigue in upcoming trials with scores like the PROMIS cancer fatigue short form [47].

For reducing risks of adverse events, the intensity prescription of the training sessions is important. The Oncology [48] and Heart Insufficiency guidelines [12] recommend moderate intensities and at the beginning low intensities [16]. Pre-exercise test are also a possibility for reducing risks for adverse events, but while imaging procedures and other tests are burdens which may actually compromise QoL, palliative medicine aims for QoL improvement [3] and patients urge for an autonomous and normal life in their last days with physical activity meaning independency and mobility [20, 49]. Therefore professionals should judge the actual need and modifications of tests before starting exercise intervention for preventing adverse events especially in cancer patients with comorbidities [16, 50].

Especially for patients with bone metastases exercise prescription is challenging but has potential for health benefits which should weighted against potential skeletal risks. For example there is no recommendation for gait training in patients with unstable bone metastases in pelvis or lower spine [26] but it is performed during classical physiotherapy to improve QoL. Nevertheless, recommendations should be considered as to which exercises and tests are safe for metastases in particular locations [51]. In general, we think a trade-off must be made between security and a normal life by explaining the risks to patients with the possibility of autonomous decisions to take risks to improve QoL.

### Retention

In our study, home-based training proved difficulties during follow-up which is in line with previous findings. Siemens et al. [40] concluded that a home-based training programme is not feasible for PCU patients after discharge because of medical problems and recruitment barriers. While we lost 3 patients during follow-up due to death or medical deterioration, medical problems were also the main reasons preventing patients from training. Another problem was the missing prescription and continuation of physiotherapy. Some patients reported that they missed the encouragement and support from physiotherapists while exercising at home. This resulted in some individuals having a smaller training volume during follow-up compared to those who trained in the hospital during follow-up. Nevertheless, we had also patients who were highly motivated and trained more than 3 times a week at home and also took care of further physiotherapy prescription. Generally, patients performed rather familiar exercises indicating the fear to fail and exceeding their physical limits. Although our results are based on a small number of follow-up patients, in combination with results of Siemens et al. [40] it appears to be important to motivate PCU patients during their hospital stay, prioritise teaching appropriate exercises and counter patients’ fears by education of physical limits and perceptive promotion. Future studies need to investigate how, and to which extend patients can be supported in continuing exercise after dismissal.

### Improvement of Quality of Life and Physical function

Although in recent exercise studies the median intervention duration was 12 weeks [10], our study data indicate that in critically ill patients improvements in QoL may already be achieved by as little as 2 weeks. While the PF of patients being able to perform pre and post PF-testing improved, patients also stated in the qualitative interviews that their fitness and mobility improved throughout the intervention. Nevertheless, 2 patients could not perform the PF-test post-exercise intervention due to disease progress resulting in a deterioration of PF. A deterioration of PF was also shown in physical QoL after 4 weeks of follow-up. This indicates a short improvement of physical QoL is possible, but 4 weeks are a long time for some terminally ill patients.

### Trial limitations and sources of potential bias

Our primary aim was the feasibility of the exercise intervention. Thus, we intended to present the challenges of an exercise intervention study in this special setting and raise awareness for this group of very ill patients. As this study comprised a single cohort design with no control group, findings in terms of the effectiveness of the intervention need to be interpreted with caution. As we only managed to enrol a small number of patients in our recruitment period, the efficacy analysis was likely underpowered and results may be subject to significant fluctuations and uncertainties. Concerning our qualitative data, the concept of data saturation cannot yet be assumed. Although numerous patients shared similar issues and experiences, each patient introduced unique aspects, and predicting their statements was not possible.

Furthermore, blinding was not possible and, therefore, recruiting and confirmation bias resulting in better results are possible. Another bias might be the highly variable daily form and symptoms of the patients while answering questions and performing PF-tests possibly resulting in an unreliability of the QoL and PF results. Moreover, we saw difficulties while testing QoL and PF in this population. Although the SF-12 is the short form of a standard QoL assessment in exercise studies for advanced tumour patients [19], for PCU patients there were too few selection options regarding the PF. The EORT-QLQ-C15 [52] is only designed for PC cancer patients but might be more suitable in future studies. Additionally, 6 exercises for PF-testing were too much for PCU patients. Likewise, weights were too heavy, and exercises did not suit all patients because of specific medical limitations. Suitable tests for our collective were the arm curl test with individual weight, 2-minute leg raise and time up and go test.

## Conclusions and future aims

The diseases and symptoms of patients on a PCU are diverse, making the screening and testing process challenging and resulting in a heterogeneous and small study population. Nevertheless, we found our 2-week exercise intervention to be feasible for PCU patients. In a period of 6 months, 10% of PCU’s cancer patients participated in the exercise intervention with good adherence rates and preferences for moderate endurance training. Even though the facilities, time constraints and a lack of well-trained personnel complicated the provision of structured exercise in our PC setting, we found the exercise intervention to be save and valued by the enrolled patients. Although our data regarding the effectiveness in improving quality of life and physical fitness are underpowered, there was a noticeable improvement in some patients. In addition, we observed that the patients were motivated to continue training during follow-up, but medical reasons prevented regular engagement and further progress of PF and QoL. The findings of QoL and PF improvement need to be confirmed and expanded by further full-scale studies planned with the guidance and learnings of our study. While the PCU setting may make exercise interventions complex, these challenges may be overcome by multicentre studies also including other PC settings (e.g. hospice and consultation services) which have a higher number of patients with a better functional status.

### Supplementary Information

**Supplementary Material 1.**

**Supplementary Material 2.**

**Supplementary Material 3.**

**Supplementary Material 4.**

**Supplementary Material 5.**

**Supplementary Material 6.**

**Supplementary Material 7.**

## Data Availability

The data sets used and/or analysed during the current study are available from the corresponding author on reasonable request.

## Abbreviations

ECOG: Eastern Cooperative Oncology Group
ET: Endurance Training
PC: Palliative Care
PCU: Palliative Care Unit
PF: Physical Function
QoL: Quality of Life
RT: Resistance Training
