# Supplementary material for: Feasibility of a physical exercise intervention for patients on a palliative care unit: a critical analysis
Source: BMC Palliat Care. 2024 Feb 28;23:58. doi: 10.1186/s12904-024-01388-5 (PMC10900709; doi:10.1186/s12904-024-01388-5)
Supplement: Supplementary file 1 — Supplementary Material 1. [file 12904_2024_1388_MOESM1_ESM.docx]

# Information for independent training

Thank you for participating in our study. For the follow-up period of 4 weeks we would like to provide you with recommendations for the continuation of the training.

**How often should I exercise?**

- 150 min of moderate-intensity endurance training (e.g. brisk walking) or 75 min of intensive training (e.g. strenuous gardening, brisk stair climbing) per week. You may choose the duration of the training sessions by yourself.
- Twice a week strength training for the upper and lower body.
- Stretching exercises two days a week.

**Where can I train?**

- There are several options
  - During physiotherapy
  - At the gym
  - At home🡪 For strength training you only need little space
  - Outdoors in nature🡪 Endurance training such as fast walking

**What materials do I need?**

- If you want to do an endurance workout outside, all you need is good footwear.
- For strength training at home, it is useful to order a resistance band (theraband) on the Internet or buy it at the medical store. You can replace dumbbells with individually filled water bottles.

**What do I have to pay attention to?**

- Exercise only when you feel well and stop exercising if you experience severe pain, dizziness or general discomfort.
- Between intensive training sessions should be a day off, or a moderate training session.
- You should slowly increase the intensity and duration.

**What exercises can I do?**

- You will find a selection of exercises in the appendix.
- For strength training, you can choose three exercises for the lower body and three exercises for the upper body.
- For endurance training, for example, you can go for a faster walk.
