# Supplementary material for: Feasibility of a physical exercise intervention for patients on a palliative care unit: a critical analysis
Source: BMC Palliat Care. 2024 Feb 28;23:58. doi: 10.1186/s12904-024-01388-5 (PMC10900709; doi:10.1186/s12904-024-01388-5)
Supplement: Supplementary file 2 — Supplementary Material 2. [file 12904_2024_1388_MOESM2_ESM.docx]

# Exercise selection for strength training at home

**On the following pages you will find sample exercises you can use for your training at home. Some exercises may also be familiar to you because you have already performed them during your stay in the palliative care unit.**

**For example, you can compile your strength training as follows:**

- **6 exercises:** (of which 3 are best for the upper body and 3 for the lower body).
  - Upper body: Arm curls, push-ups, butterfly, or similar.
  - Lower body: Lunges to the side, heel raises, standing up from a seated position, or similar.
- **Start**: (warm-up program as you did during the training sessions in the palliative care unit).
  - Examples: Circle arms, circle shoulders, circle feet, walk a few steps.
- **Training:**
  - Biceps curls 8-12 repetitions

3 sets, i.e. 3x 8-12 repetitions

Break

- - Push-ups 8-12 repetitions

3 sets, i.e. 3x 8-12 repetitions

Break

- - ...
- **Cool down**: 3 min stretching
  - Stretching exercises as learned in physiotherapy

**Exercises for the upper body:**

Additional weights can include water bottles to replace dumbbells, resistance bands, and a backpack to increase the difficulty.

| **Exercise name** | **Description** | **Basic exercise** | **Difficulty 1** | **Variation/ Difficulty 2** |
| --- | --- | --- | --- | --- |
| **Arm bends**  **(Biceps curls)** | Bend the arm. Make sure that the elbow remains close to the body. The upper body is upright or slightly bent forward with a straight back. The exercise can be done standing or sitting. A water bottle, for example, can serve as a weight. | Sitting with light weights | Standing, choose weights heavier and heavier | With resistance band in sitting or standing position |
| **Arm stretches (triceps extensions)** | Stretch the arm. The arm is brought overhead so that the elbow points upwards. The forearm is then stretched and bent alternately. The upper body is upright. The back remains straight (no hollow back). The exercise can be done standing or sitting. A water bottle, for example, can serve as a weight. | Sitting | Standing with band or weights | Backwards against bench/chair |
| **Butterfly** | With a resistance band or an additional load (e.g. Water bottles, smaller dumbbells), the arms are stretched out to the side at the level of the rib cage and brought together again. The upper body is upright, the back remains straight. | Sitting | Standing |  |
| **Reverse Butterfly** | Reverse butterfly only works with a resistance band. This lies across the back at the shoulder blades. Now both ends of the band are brought together with stretched arms in front of the chest.  The upper body is upright, the back remains straight. | Sitting | Standing |  |
| **Shoulder**  **Press /side lift** | Starting position: Weights are brought up to shoulder height and elbows point down, slightly forward. From here, arms are stretched straight up toward the ceiling (knuckles are the highest point, wrist not "bent") before lowering back to shoulder height. | End position: stretch arms above head | End position: arm stretched to the side at shoulder level | Both arms stretched sideways at shoulder height |
| **Rowing** | Version 1:  A resistance band is placed around the feet. Then tighten the arms by pulling the elbows past the ribs. The upper body is upright, the back remains straight (no hollow back).  Version 2:  Bending forward rowing by lowering the upper body (keeping the back straight). The additional load is now lifted vertically upwards by pulling the elbows towards the ceiling. As weight can serve e.g. A water bottle | Version 1:  Sitting on the floor | Version 2:  Move only the arms |  |
| **Push-up** | The entire body is stretched as much as possible. Under tension, the upper body is lowered to the floor by bending the arms. The elbows are close to the body (not away to the side). After lowering the upper body, the arms are stretched again until the starting position is reached. | Standing against wall. Heavier the further the feet are from the wall | On bench / stool | In support, for easier variant with knees on the floor |

**Exercises for the lower body**

Additional weights can include water bottles to replace dumbbells, resistance bands and a backpack to increase difficulty

| **Exercise name** | **Description** | **Basic exercise** | **Difficulty 1** | **Difficulty 2** |
| --- | --- | --- | --- | --- |
| **Squat** | Lower the buttocks backwards. Make sure that your back is straight. A stool can be placed under the buttocks for safety. Please make sure that the knees do not come close to each other. | Getting up from the chair and sitting down again, preferably without hands | Squat from a standing position. Possibly with weights | With resistance band around the thighs |
| **Torso bend** | Tilt the upper body forward, making sure that the back remains stretched (straight). | Without resistance band | With resistance band | With dumbbells/ water bottles |
| **Lunge forward/**  **Backward** | Large lunge forward or backward. Make sure back is straight, upper body remains upright, gaze is straight ahead. Knee lowers to the floor. Both knees are bent | Without resistance bands/weights | With weights in the hands | With resistance band around the thighs |
| **Lunge sideways**  **(harder than forward and backward)** | Lunge alternately to the left and right. Ensure a straight back, upper body remains upright, gaze is directed straight ahead. | Vary depth | With additional weights in the hands: (dumbbells or water bottles) |  |
| **Step-up** | Change steps onto an elevation. Upper body remains upright, gaze is directed straight ahead. |  | Increase step height | Use additional weights |
| **Leg lift** | Lifting the legs to the side in a standing position or upwards from a lying position. | Lying down, alternately raise and lower legs upwards. | Lateral lift without resistance band | With resistance band |
| **Tighten knees/**  **Stretches** | Tightening the knees while standing or sitting or lying down. | Lying with or without belt | Standing with or without a band, supporting yourself against the wall |  |
| **Heel**  **Lift** | Heel lift through toe stand. For stabilization can be used the wall | Without additional weight | With additional weight | Single leg with additional weight |
