# Supplementary material for: Feasibility of a physical exercise intervention for patients on a palliative care unit: a critical analysis
Source: BMC Palliat Care. 2024 Feb 28;23:58. doi: 10.1186/s12904-024-01388-5 (PMC10900709; doi:10.1186/s12904-024-01388-5)
Supplement: Supplementary file 3 — Supplementary Material 3. [file 12904_2024_1388_MOESM3_ESM.docx]

# PallTrain – Exercise Intervention

# GENERAL

- Intervention duration: 2 - 3 weeks
- ECOG 1 -3 included, subdivision of training according to classification
- Training times daily from Monday to Friday
- Global rest time after intensive sessions, i.e. RPE 7-9 (strength and endurance)

# ENDURANCE TRAINING

- Bike ergometer
- Hand crank ergometer
- 10 meters walking distance on the corridor
- Progression

## Moderate and continuous endurance training (RPE: 4-6).

**Training session 1-3**

**WARM-UP: 3 minutes without load**

ECOG-3: 2 sets of 5 minutes (RPE 4-6) with break of 2 minutes (RPE 1)🡪 12 minutes | 18 minutes

ECOG-2: 3 sets of 5 minutes (RPE 4-6) with break of 2 minutes (RPE 1)🡪 19 minutes | 25 minutes

ECOG-1: 4 sets of 5 minutes (RPE 4-6) with break of 2 minutes (RPE 1)🡪 26 minutes | 32 minutes

**COOL-DOWN: 3 minutes without load**

**Training session 4-6**

**WARM-UP: 3 minutes at 20% load of the last unit**

ECOG-3: 3 sets of 5 minutes (RPE 4-6) with break of 2 minutes (RPE 1)🡪 19 minutes | 25 minutes

ECOG-2: 4 sets of 5 minutes (RPE 4-6) with break of 2 minutes (RPE 1)🡪 26 minutes | 32 minutes

ECOG-1: 3 sets of 7 minutes (RPE 4-6) with break of 2 minutes (RPE 1)🡪 25 minutes | 31 minutes

**COOL-DOWN: 3 minutes without load**

**Training session >6**

**WARM-UP: 3 minutes at 20% load of the last unit**

ECOG-3: 4 sets of 5 minutes (RPE 4-6) with break of 2 minutes (RPE 1)🡪 26 minutes | 32 minutes

ECOG-2: 3 sets of 7 minutes (RPE 4-6) with break of 2 minutes (RPE 1)🡪 25 minutes | 31 minutes

ECOG-1: 2 sets of 10 minutes (RPE 4-6) with break of 2 minutes (RPE 1)🡪 22 minutes | 28 minutes

**COOL-DOWN: 3 minutes without load**

## Intensive endurance training in the form of interval training (RPE: 7-9).

**Training session 1-3 (1 : 3 ratio):**

**WARM-UP: 3 minutes without load**

ECOG-3: 4 sets of 30 seconds (RPE 7-9) with rest of 90 seconds (RPE 1)🡪 8 minutes | 14 minutes

ECOG-2: 6 sets of 30 seconds (RPE 7-9) with rest of 90 seconds (RPE 1)🡪 12 minutes | 18 minutes

ECOG-1: 8 sets of 30 seconds (RPE 7-9) with rest of 90 seconds (RPE 1)🡪 16 minutes | 22 minutes

**COOL-DOWN: 3 minutes without load**

**Training session 4-6 (1 : 2 ratio):**

**WARM-UP: 3 minutes without load**

ECOG-3: 4 sets of 60 seconds (RPE 7-9) with rest of 120 seconds (RPE 1)🡪 12 minutes | 18 minutes

ECOG-2: 6 sets of 60 seconds (RPE 7-9) with rest of 120 seconds (RPE 1)🡪 15 minutes | 21 minutes

ECOG-1: 8 sets of 60 seconds (RPE 7-9) with rest of 120 seconds (RPE 1)🡪 18 minutes | 24 minutes

**COOL-DOWN: 3 minutes without load**

**Training session >6 (1 : 1 ratio):**

**WARM-UP: 3 minutes without load**

ECOG-3: 4 sets of 60 seconds (RPE 7-9) with rest of 60 seconds (RPE 1)🡪 8 minutes | 14 minutes

ECOG-2: 6 sets of 60 seconds (RPE 7-9) with rest of 60 seconds (RPE 1)🡪 12 minutes | 18 minutes

ECOG-1: 8 sets of 60 seconds (RPE 7-9) with rest of 60 seconds (RPE 1)🡪 16 minutes | 22 minutes

**COOL-DOWN: 3 minutes without load**

# STRENGTH TRAINING

- Goal 3 exercises each for upper and lower body
- In case of bone metastases the table “M3EP for PCa with bone metastases” from Galvao et al.(1) was used to select appropriate exercises

### Materials

- Dumbbells
- Drawstrings
- Quilting board
- Cone
- Chair

### Exercise catalog

**Lower body**

- Squat
- Lunge forward/backward
- Lunge to the side
- Tap up on tap board
- Get up from sitting
- Heel lift

**Upper body**

- Biceps Curls
- Triceps Kickbacks
- Shoulder press
- Rowing
- Trunk bend (standing)
- Pushups with elevation

## Moderate strength training (RPE: 4-6)

**Training session 1-3:**

**WARM-UP: 3 minutes of dynamic stretching**

ECOG-3: 4 exercises, 2 sets of 15-20 repetitions (RPE 4-6); rest 2 minutes between sets and 1 minute rest between exercises🡪 30-40 repetitions per exercise.

ECOG-2: 4 exercises, 3 sets of 12-15 repetitions (RPE 4-6); rest 2 minutes between sets and 1 minute rest between exercises🡪 36-45 repetitions per exercise.

ECOG-1: 4 exercises, 3 sets of 10-12 repetitions (RPE 4-6); rest 2 minutes between sets and 1 minute rest between exercises🡪 30-36 repetitions per exercise.

**COOL-DOWN: 3 minutes static stretching**

**Training session 4-6:**

**WARM-UP: 3 minutes of dynamic stretching**

ECOG-3: 6 exercises, 2 sets of 15-20 repetitions (RPE 4-6); rest 2 minutes between sets and 1 minute rest between exercises🡪 30-40 repetitions per exercise.

ECOG-2: 6 exercises, 3 sets of 12-15 repetitions (RPE 4-6); rest 2 minutes between sets and 1 minute rest between exercises🡪 36-45 repetitions per exercise.

ECOG-1: 6 exercises, 3 sets of 10-12 repetitions (RPE 4-6); rest 2 minutes between sets and 1 minute rest between exercises🡪 30-36 repetitions per exercise.

**COOL-DOWN: 3 minutes static stretching**

**Training session >6:**

**WARM-UP: 3 minutes of dynamic stretching**

ECOG-3: 6 exercises, 3 sets of 12-15 repetitions (RPE 4-6); rest 2 minutes between sets and 1 minute rest between exercises🡪 36-45 repetitions per exercise.

ECOG-2: 6 exercises, 4 sets of 10-12 repetitions (RPE 4-6); rest 2 minutes between sets and 1 minute rest between exercises🡪 40-48 repetitions per exercise.

ECOG-1: 6 exercises, 4 sets of 8-10 repetitions (RPE 4-6); rest 2 minutes between sets and 1 minute rest between exercises🡪 32-40 repetitions per exercise.

**COOL-DOWN: 3 minutes static stretching**

## Intensive strength training (RPE: 7-9)

**Training session 1-3:**

**WARM-UP: 3 minutes of dynamic stretching**

ECOG-3: 4 exercises, 2 sets of 8-10repetitions (RPE 7-9); break of 2 minutes between sets and 1 minute break between exercises🡪 16-20repetitions per exercise

ECOG-2: 4 exercises, 3 sets of 8-10 repetitions (RPE 7-9); rest 2 minutes between sets and 1 minute rest between exercises🡪 24-30 repetitions per exercise.

ECOG-1: 4 exercises, 3 sets of 5-8 repetitions (RPE 7-9); rest 2 minutes between sets and 1 minute rest between exercises🡪 15-24 repetitions per exercise.

**COOL-DOWN: 3 minutes static stretching**

**Training session 4-6:**

**WARM-UP: 3 minutes of dynamic stretching**

ECOG-3: 6 exercises, 2 sets of 8-10 repetitions (RPE 7-9); rest 2 minutes between sets and 1 minute rest between exercises🡪 16-20 repetitions per exercise.

ECOG-2: 6 exercises, 3 sets of 6-8 repetitions (RPE 7-9); rest 2 minutes between sets and 1 minute rest between exercises🡪 18-24 repetitions per exercise.

ECOG-1: 6 exercises, 4 sets of 3-5 repetitions (RPE 7-9); rest 2 minutes between sets and 1 minute rest between exercises🡪 12-20 repetitions per exercise.

**COOL-DOWN: 3 minutes static stretching**

**Training session >6:**

**WARM-UP: 3 minutes of dynamic stretching**

ECOG-3: 6 exercises, 4 sets of 6-8 repetitions (RPE 7-9); rest 2 minutes between sets and 1 minute rest between exercises🡪 24-32 repetitions per exercise.

ECOG-2: 6 exercises, 4 sets of 3-5 repetitions (RPE 7-9); rest 2 minutes between sets and 1 minute rest between exercises🡪 12-20 repetitions per exercise.

ECOG-1: 6 exercises, 5 sets of 3-5 repetitions (RPE 7-9); rest 2 minutes between sets and 1 minute rest between exercises🡪 15-25 repetitions per exercise.

**COOL-DOWN: 3 minutes static stretching**

1. Galvão DA, Taaffe DR, Spry N, Cormie P, Joseph D, et al. 2018. Exercise Preserves Physical Function in Prostate Cancer Patients with Bone Metastases. *Medicine & Science in Sports & Exercise* 50:393-9
