# Supplementary material for: Feasibility of a physical exercise intervention for patients on a palliative care unit: a critical analysis
Source: BMC Palliat Care. 2024 Feb 28;23:58. doi: 10.1186/s12904-024-01388-5 (PMC10900709; doi:10.1186/s12904-024-01388-5)
Supplement: Supplementary file 4 — Supplementary Material 4. [file 12904_2024_1388_MOESM4_ESM.docx]

# Material list

| **Name material** | **Kg** | **Quantity** |
| --- | --- | --- |
| Step board |  | 1 |
| Kettlebells | 4 | 2 |
|  | 5 | 2 |
|  | 6 | 2 |
|  | 8 | 2 |
|  | 9 | 2 |
| Dumbbells | 1 | 2 |
|  | 2 | 2 |
|  | 3 | 2 |
|  | 4 | 2 |
|  | 5 | 2 |
|  | 6 | 2 |
|  | 7 | 2 |
|  | 10 | 2 |
| Short loop band | Mint green | 10 |
|  | Pink | 10 |
|  | Purple | 10 |
| Long loop band | Red | 2 |
|  | Gray | 2 |
|  | Purple | 2 |
|  | Green | 2 |
| Sling Trainer |  | 1 |
| Bicycle ergometer |  | 1 |
| Hand crank ergometer |  | 1 |
