# Supplementary material for: Feasibility of a physical exercise intervention for patients on a palliative care unit: a critical analysis
Source: BMC Palliat Care. 2024 Feb 28;23:58. doi: 10.1186/s12904-024-01388-5 (PMC10900709; doi:10.1186/s12904-024-01388-5)
Supplement: Supplementary file 5 — Supplementary Material 5. [file 12904_2024_1388_MOESM5_ESM.docx]

Semi-structured interview guides

# Qualitative interview

**General instructions**

- First questions especially open and let the patient talk until the end, while leading the patient just slightly. Take notes in case you want to refer to anything.

**Pre-information**

Before the interview the interviewer collected information about the conducted training sessions (kind, number) and informed the patients about the following points:

- Introduction of the interviewer
- Duration and procedure of the interview
- Thanking the patient for participating
- Asking the patient: “If you do not want to answer questions or do not want to continue the interview you can do so at any time.”
- Anonymous data: Audio data of the interview will be transcribed, anonymously processed and assembled. Results will be processed as part of the study. Recordings will be deleted from all other end-devices after completion of the study.
- Asking the patient: “Do you agree that I record our interview for evaluation purposes? I can assure you that anonymity will be maintained and that it will not be possible to draw any conclusions about you. For this reason, I will not mention your name during the interview.”
- Giving the patient the opportunity to ask further questions

**Entry question**

- **Which role had sports in your life?**
  - What kind of sports did you do?
  - How often?
  - Changes during your whole life?
  - Other hobbies?

**Leading questions:**

- **Which expectations did you have of the exercise intervention study?**
  - Were your expectations fulfilled? What led to expectations being fulfilled?
- **How did you like the exercise intervention?**
  - Any problems?
  - Which training type did you like the most? 🡪 Which exercises did you like?
  - Suggestions for improvement?
  - What do you think are the reasons for perceived positive/negative effects?
- **Have you experienced/felt any changes due to the exercise training?**
  - E.g. fatigue, pain, mood
- **What was your highest barrier to motivate yourself?**
- **What do you think about the plan to establish an exercise training program into standard palliative care?**
  - Would you recommend our program to other patients?
- **Do you want to add anything?**
  - Suggestions for improvement

**Summary and Thanks**

**Notes after the interview**

- General impressions of the patient
- Main points of the interview
- Deviations from the interview guide

# Telephone interview

Conducted after first and fourth week of follow-up with the SF12 questionnaire.

- **How are you?**
- **How many training sessions did you do in the last week/weeks?**
  - What kind of training/exercises did you do?
  - Where did you train?
- **What was the highest barrier to you in order to train in the last weeks? (Important question)**
  - In your opinion what would reduce those barriers?
- **If training sessions conducted: How did you feel after the training sessions?**
  - Which changes have you experienced?

(e.g. mood, motivation, pain, fatigue)
