# Supplementary material for: Feasibility of a physical exercise intervention for patients on a palliative care unit: a critical analysis
Source: BMC Palliat Care. 2024 Feb 28;23:58. doi: 10.1186/s12904-024-01388-5 (PMC10900709; doi:10.1186/s12904-024-01388-5)
Supplement: Supplementary file 6 — Supplementary Material 6. [file 12904_2024_1388_MOESM6_ESM.docx]

**Summary table qualitative interviews follow-up**

**Training 1 week follow-up**

| **Frequency of training** | **Type of training** | **Reasons/ hurdles why not trained** | **Actions to overcome the hurdles** |
| --- | --- | --- | --- |
| - Continued on ward and trained almost always when physiotherapist came | - gait training done with physiotherapists | - Fatigue has prevented training |  |
| - Made four training sessions in the last week. | - Intensive training with physiotherapy (Theraband and strengthening arms and legs) and independent training with arms on the bed-ergometer. | - Flatulence and problems with bowel movements prevented training. Training had to be postponed. |  |
| - Daily 10 min theraband training | - Known theraband exercises performed daily. - Exercise selection not used. - Getting out of bed exercises although it is difficult made. | - The biggest hurdle is the psychological situation and that not everything works out as imagined. Then rest necessary | - The biggest hurdle is the psychological situation and that not everything works out as imagined. Then rest necessary |
| - Trained a total of eight times, four times in the morning and four times in the afternoon | - Strength training performed in a sitting and lying position. 3 exercises 3 sets of 12 repetitions. - Stretching legs, lifting under pressure. - Stair climbing as endurance training | - Gastrointestinal flu has come in between otherwise no hurdles |  |
| - No training performed |  | - No motivation | - Motivation to train by someone actively approaching him and asking if he wants to train. - Otherwise, no idea how motivation can be achieved. Setting alarm clock would not help |
| **Training 4 weeks follow-up** | | | |
| **Frequency** | **Type of training** | **Reasons/ hurdles why not trained** | **Measures to overcome the hurdles** |
| - No workouts done since dismissal home |  | - Getting up alone no longer possible and training alone is boring. Physio has not yet been prescribed | - Need physical therapy, but it has not been prescribed. - No doctor contact available. - Believes with physiotherapy it would go better - Assistance with training would be needed |
| - Did two workouts in the last three weeks with physio and continued to do a lot of independent strength training without physio. Physiotherapist comes twice a week for training. | - Intensive physio training and independent strength training for legs and arms. | - Abdominal problems have prevented training and theraband is lost. Must first be procured new | - therabands have disappeared. Therefore, no training for arms possible. Physiotherapist wants to get new bands |
| - Three, Four times a week still trained. Number of training sessions depends on the conditions of the day. Daily training is not possible. | - Did stand-up exercises and different exercises. - Not all exercises as in the clinic - Also continued to do theraband training. | - Reasons for not exercising: Fatigue and limpness | - no further training support necessary. - What I can do is okay |
| - From discharge on 03.02 to 21.02 exercised every day and additionally cycled on ergometer on good days. | - Strength training - stair climbing - stretching exercises - cycling. | - Biggest hurdle shortness of breath. Severe with exertion | - Breathing training for shortness of breath often performed |
| - Four to five training sessions in the last three weeks | - Been running in the park | - No motivation | - Must force himself to go out |

**Experience with exercise intervention follow-up 1 week**

| **Experience with sports therapy after discharge** | **Changes due to exercise therapy** | **Questions** | **Positive Experiences** | **Goals/ motivation** |
| --- | --- | --- | --- | --- |
| - Gait training is a necessary evil | - Tired and sleepy after training. No change in pain, motivation, mood after training. |  |  |  |
| - Physical therapy in the short-term care already registered. Will then continue training - Got muscle soreness in chest area during theraband workout. - Sore muscles better again. - Ergometer training was possible again | - Has noticed that his body can still move and it is possible to train muscles and endurance. - More strength in arms through training. - Legs still slightly worse than arms. - Mood, motivation and fatigue have not changed. Good sleep quality despite waking up several times because also slept during the day |  | - Strength in arms is okay. Mobilization in and out of the chair is possible - Felt healthily exhausted after workout. Felt very good - Nice feeling in arms after intense workout |  |
|  | - Felt good after workout but physically exhausted | - Difficulty finding safe place for theraband training | - Paraesthesia in toes improved, questionable whether through sports therapy |  |
|  | - Notices that strength comes back through sports | - One week until dismissal from hospital. - Then wants to continue training at home and wonders if he still has to do something for it. - Training at home possible without problems with exercise bike and help from the wife. | - Is proud of himself to have still managed something despite the poor prognosis. | - The goal is to get fitter again. It was better before surgery. Only after surgery it got so bad. - Great goal to get as fit as before surgery |

**Experience with exercise intervention follow-up 4 weeks**

| **Experience with sports therapy after discharge** | **Changes due to exercise therapy** | **Positive Experiences** | **Goals/ motivation** |
| --- | --- | --- | --- |
| - Took care of physical therapy himself because he wanted some - Physiotherapy was not prescribed and is not covered by health insurance | - Endurance has improved. Standing and going to the toilet possible without shortness of breath and gasping for breath. - No changes felt in mood, fatigue, motivation due to sports. Sport does not influence sleep rhythm. - Sleep is disturbed by medication.  If medication is taken correctly, sleep is good especially after sports. - Yesterday abdominal problems affected very much - Due to sport he is already stronger and has built leg muscles. - Improvements need time. Changes need time | - Physical therapist is really good, bends him in the right way. He even sweats during training - Feeling good after the workout: Loosened up and strengthened - Through sports he is already stronger and has built leg muscles. Improvements take time. Not everything can be done within three weeks - Glad to be able to stand again without problems with holding. | - First goal to drive with the wheelchair he has achieved - Is firmly convinced to achieve the second big goal, to walk again, in next months due to rehab |
| - Standing up exercises and getting up from a squat position is very strenuous. Rollator is necessary to get up | - Hoped to become more stable through training. But this is not the case. - Physically exhausted after training - Pain or mood not changed by training |  | - Motivated to train - Does not want to run around as a tailcoat and still do something. - Hoped to become more stable through training. But is not the case |
|  | - Strengths return and movements of the arms and legs improve. | - Felt well after workout - Fatigue at home due to exercise became better - No more need for a nap |  |
|  | - After the workout felt quite normal - Muscle soreness in the legs after training - No change in fatigue, pain, mood |  |  |

This file was originally in German language. For the purpose of publication the file was translated with DeepL and post edited(1).

1. DeepL. *DeepL Translator*. <https://www.deepl.com/translator>
