# Supplementary material for: Feasibility of a physical exercise intervention for patients on a palliative care unit: a critical analysis
Source: BMC Palliat Care. 2024 Feb 28;23:58. doi: 10.1186/s12904-024-01388-5 (PMC10900709; doi:10.1186/s12904-024-01388-5)
Supplement: Supplementary file 7 — Supplementary Material 7. [file 12904_2024_1388_MOESM7_ESM.docx]

| **Role of sport in life** | **Frequency of sports in life** | **Sports** |
| --- | --- | --- |
| - Never done sports | - Rarely walked - No sport even as a child | - Walking |
| - Sport was the most important thing in the world | - Played tennis almost every day until a few weeks ago | - Tennis - Soccer - A lot more |
| - Very important role - Martial arts trainer license - The goal of the training was fitness and weight maintenance | - After trainer license little practiced martial arts himself - During work 3-4 times fitness study 1-1.5h for 25 years consistently | - Martial Arts - Gym |
| - Very important in life - Got secondary because of work | - During work always moved a lot but no longer explicitly performed sports. - Before 2 times per week | - Athletics, cycling, - Physical training through work |
| - Sport had no big role in life | - Twice a week kisa-training and cycling depending on the weather. - Skiing earlier - Did little sports as a child because parents did not have a car | - Kisa-training - Cycling |
| - High importance and great role - Always been a movement person | - Before Corona two-three times a week kisa-training and twice a week dancing in social circle - Independent practice of the learned dance steps according to Kisa-Training - Walked a lot and did errands by bike - During Corona trained little | - Kisa-trainig - dance sport, independent training of dancing steps - Walking and errands by bike |
| - A lot of sports in life. - Played darts professionally with numerous titles | - Played a lot of sports in life - For 30 years, regularly exercised up to four times a week and played darts professionally. Then trained daily | - Soccer - Handball - Skittles - Dart |
| - Had great role in life | - Played two to four times a week | - Mainly ball sports such as table tennis, badminton, tennis, soccer played |
| - Profession came first. Sports was subordinate | - Moves a lot during work and additionally exercises 5-10 min every day | - Endurance training on ergometer and strength training at home - Walking during work |
| - No major role in life | - Sometimes more, sometimes less sports in the course of life. - Made an effort to do sports, but rarely had time for it | - Hiking, tennis, and other sports at different times of life |
| - Had great role in life | - 3-4 times a week. | - Soccer, gym, - Depending on the day also running |

**Summary table qualitative interviews post intervention**

| **Exercise intervention expectations** | **Positive experience with exercise intervention** | **Negative experience with exercise** |
| --- | --- | --- |
| - Expectation of sports therapy: Get up again and moving on |  | - Did not like therapy because it did not bring anything and was always the same - No fun at all |
| - Maybe it will get a little better again |  |  |
|  | - Therapy was very good.  Always addressed problems  intensively and selectively - Thanks for offer of the study - Through training   - Feels better physically   - Great development of strength in both arms and legs/abdomen   - Independent relocation possible again   - Mentally helped to clear the mind   - Gained agility | - Standing up sometimes did not work. Setback because strength and coordination were not yet back again |
| - No expectations - Objectives: Gather strength, become more agile to be support for wife. | - Therapy has been very good - Positive feedback due to daily increase in functionality - Built muscles and become more stable - Standing now possible | - Rapid muscle growth has led to displacement of fat nodules that have pressed on nerves/tendons |
| - Become more mobile again | - Did very well  before therapy fear of climbing stairs now possible again |  |
| - Therapy supports intensive strength building and improvement of endurance | - Has liked therapy well but it was also very exhausting - Great run and benefited of it - Strength significantly improved - Endurance improved also a little - Walking without rollator possible again - More stable on the legs |  |
|  |  | - Rather had problems - Mind circling and brooding through sports therapy |
| - Stay physically fit, have fun, socialise with other people | - Has liked therapy well | - Different types of training in a row too exhausting - More often overestimated and reached and exceeded load limit |
| - Getting fitter again | - Feels fitter and has achieved something due to training - Possibility to stay in motion, to work with people and to get into conversation - Would like to continue training |  |
| - Didn't have high expectations for exercise therapy because didn't expect there to be any - Pleased to hear there is an opportunity to participate in sports therapy. | - Liked it well, could have used it even more |  |
| - Had no expectations / imagination | - Liked exercise therapy well - Change in everyday life through movement - Felt good after training | - Muscle soreness in legs |

| **Training type** | **Neutral experience** | **Reasons/hurdles why not trained in clinic** |
| --- | --- | --- |
| - Only gait training done | - Therapy has brought nothing - No changes or problems | - Did not give any hurdle to motivate for training |
| - Own movement has the most impact - Hand crank training was good because it was more intense than just climbing stairs | - Training does not bring much - No change experienced through training with physiotherapy | - No hurdle to move. Moves always |
| - Hand crank ergometer was best | - No change in mood due to therapy | - The biggest hurdle for joining was accepting the own condition. |
| - Physiotherapists have chosen gait training to treat specific medical problems | - No problems, no change in mood/motivation | - No motivation hurdle. As long as I breathe, I live. That is the motivation. No one to tell you what to do |
| - Endurance and strength training were both okay | - No change of fatigue or mood | - The biggest hurdle was finding motivation to train at all - Not dared to do anything for a year because of back pain. Big surmounting to start again |
| - Tried all types of workouts and all were fun. Variety is good | - Effort clearly noticed compared to before the disease. - No change in pain - Endurance has not yet improved so much |  |
|  | - Sport was okay has brought little improvement |  |
| - Endurance training as well as strength training |  |  |
| - Had done mainly endurance training but can also imagine strength training | - No problems - No change in pain because none present and mood was unchanged good | - As soon as there is a chance of success, there is immediate motivation. There are no hurdles |
|  | - Does not make sense to talk about what changes sports therapy. Everything changes something and every situation is different. | - Gave no hurdle to motivate themselves |
| - Endurance training has liked best | - no change in fatigue, pain, mood |  |

| **Reasons/hurdles why not trained in clinic** | **Reasons for participation in exercise intervention** | **Suggestions for improvement** |
| --- | --- | --- |
| - Did not give any hurdle to motivate for training |  |  |
| - No hurdle to move. Moves always | - Exercise in the ward he would like to do and always try out | - No suggestions for improvement |
| - The biggest hurdle for joining was accepting the condition. | - Reasons for participation were to have fun and variety, even if it is exhausting. Effort is part of it | - Devices not so optimal  hand crank ergometer heavy and bulky - No bed-ergometer for the legs available has bought himself one. - Physical therapists were great, no improvement needed - Fitness room for patients would be good - Specific training for medical problems - Development of a specific training plan for each patient so that the patient can adjust to what is being done. - Paying attention to balanced training that is adapted to the patient's problems |
| - No motivation hurdle. As long as I breathe, I live. That is the motivation. No one should tell you what to do | - Does not want to lie around - Become more independent to help with care - Wants to support his wife - Participation in therapy was not a question | - No improvement for physiotherapists. - Doctors only look from their point of view and try to interfere |
| - Biggest hurdle was motivation to train at all. - Not dared to do anything for a year because of back pain. Big hurdle to start again | - Aims to gain mobility again | - Addressing problem areas and individual wishes more and re-evaluate several times |
| - As soon as there is a chance of success, there is immediate motivation. There are no hurdles | - Hope to build strength and stability to get back home and climb stairs there | - Increase frequency - Keep patients aware of all the ways they can exercise and offer different workouts |
| - Gave no hurdle to motivate themselves | - Getting fitter again after surgery. - Motivation boost when asked about participation | - No suggestions for improvement |
| - Gave no hurdle to motivate for sports | - Sport is important | - No suggestions for improvement |

| **Sports therapy as part of palliative therapy** | **Reason for recommendation** |
| --- | --- |
| - Does not think anything of it |  |
| - He would like it | - Exercise training is a good thing, even if you do not really want to |
| - Is positive towards it - clear recommendation to others | - Everyone should have courage because it is good |
| - Is critical for every patient - Always depends on patients. - Would not recommend it. Everybody must decide for himself |  |
| - Would be good for patients and would recommend it to others |  |
| - Would be good and important for everybody. Has already recommended it to others | - Thinks that it can not hurt anyone and is good and important for all |
| - Well imaginable | - Get other ideas |
|  | - Sport makes sense |

This file was originally in German language. For the purpose of publication the file was translated with DeepL and post edited(1).

1. DeepL. *DeepL Translator*. <https://www.deepl.com/translator>
